# Physical and electrochemical properties of synthesized carbon nanotubes [CNTs] on a metal substrate by thermal chemical vapor deposition

**DOI:** 10.1186/1556-276X-7-61

**Published:** 2012-01-05

**Authors:** Yong Hwan Gwon, Jong Keun Ha, Kwon Koo Cho, Hye Sung Kim

**Affiliations:** 1 School of Materials Science and Engineering, i-Cube Center, Engineering Research Institute (ERI), Gyeongsang National University, 900 Gajwa-dong, Jinju 660-701, South Korea; 2 Department of Nanomaterials Engineering, College of Nanoscience & Nanotechnology, Pusan National University, 50 Cheonghak-ri Samnangjin, Miryang-si, 627-706, South Korea

**Keywords:** carbon nanotubes, metal substrate, electrochemical property, Au layer, catalyst

## Abstract

Multi-walled carbon nanotubes were synthesized on a Ni/Au/Ti substrate using a thermal chemical vapor deposition process. A Ni layer was used as a catalyst, and an Au layer was applied as a barrier in order to prevent diffusion between Ni and Ti within the substrate during the growth of carbon nanotubes. The results showed that vertically aligned multi-walled carbon nanotubes could be uniformly grown on the Ti substrate (i.e., metal substrate), thus indicating that the Au buffer layer effectively prevented interdiffusion of the catalyst and metal substrate. Synthesized carbon nanotubes on the Ti substrate have the diameter of about 80 to 120 nm and the length of about 5 to 10 μm. The Ti substrate, with carbon nanotubes, was prepared as an electrode for a lithium rechargeable battery, and its electrochemical properties were investigated. In a Li/CNT cell with carbon nanotubes on a 60-nm Au buffer layer, the first discharge capacity and discharge capacity after the 50th cycle were 210 and 80 μAh/cm^2^, respectively.

## Introduction

Carbon nanotubes [CNTs] have recently attracted considerable attention as promising electrode materials for lithium-ion batteries due to their exceptional structure [1]. CNTs have been studied extensively and found to be promising as a new nanoscale material for a variety of potential applications owing to their excellent electrical properties, mechanical strength, and high resistance to chemical attacks [2-6]. Among various processes of CNT synthesis, chemical vapor deposition is particularly promising because CNTs can be synthesized with high yield and high purity. This process also allows direct growth of CNTs on substrates with a selective area and vertical alignment. In order to fabricate various nanodevices, controlled growth of CNTs on suitable substrates is a key issue. For such practical applications, a high-quality contact between the CNTs and conductive substrates is essential to provide an *in-situ *electrical connection for individual CNTs. Non-conductive substrates, such as silicon wafers and quartz plates, have thus far usually been used to synthesize CNTs. However, in applications such as electrodes, it is desirable to grow CNTs directly on conductive substrates, especially metallic substrates. The growth of CNTs directly on metallic substrates also resolves the problem of adhesion of nanotube layers and fulfills the requirement of substrate electroconductivity [7,8]. Such a one-step method is also advantageous in electrode preparation for lithium battery application. When metal is used as a substrate for CNT growth by CVD, a buffer layer should be deposited between the metal substrate and catalyst film (e.g., Ni, Co, or Fe) to prevent interdiffusion and interaction between the catalyst and substrate. Also, a conductive buffer layer must be used to establish electroconductivity between the CNTs and the substrate after synthesis of CNTs.

In this work, a Ni/Au/Ti substrate was used to grow vertically aligned CNTs by thermal chemical vapor deposition with acetylene gas as a carbon source. Au was applied as a barrier layer to prevent interdiffusion between the Ni layer, employed as a catalyst material, and the Ti substrate, and the effect of the Au layer according to thickness on the CNT growth was investigated. Electrochemical properties of a Ti substrate covered with CNTs were also evaluated.

### Experimental details

CNTs were grown on Ni-coated Ti substrates with an Au buffer layer by TCVD. A catalyst Ni layer with a thickness deposition of 10 nm and buffer layers with thicknesses of 20, 40, or 60 nm were deposited using a radiofrequency magnetron sputtering system. To produce Ni particles, NH_3 _gas was introduced at 873 K for 15 min. After the pretreatment processes, CNTs were grown at 1173 K for 10 min using a mixture of acetylene and ammonia flowed to the reactor chamber. The morphology, density, and quality of the CNTs were analyzed using a field emission scanning electron microscope [FE-SEM] (XL30 S FEG, Philips, Amsterdam, The Netherlands) and a high-resolution micro-Raman spectrometer (Ar^+ ^laser, 514 nm, LabRAM HR800 UV, HORIBA, Japan). One molar of LiPF_6 _dissolved in a mixture of ethylene carbonate and diethyl carbonate (1:1 by volume) was employed as a liquid electrolyte to evaluate the electrochemical properties. To investigate the electrochemical properties, a test cell was assembled in a stainless steel case (Swagelok^®^, Seoul, South Korea) by stacking, in the following order: a lithium foil, a polypropylene separator (Celgard 2400, Cheongwon-gun, South Korea) containing the liquid electrolyte, and a CNT electrode. The cell was tested in a voltage range of 0.01 to 2.0 V with a current of 50 μA. Charge/discharge tests were carried out via the galvanostatic method using a WBCS3000 battery cycler (WonATech, Seoul, South Korea).

## Results and discussion

**Figure 1:**
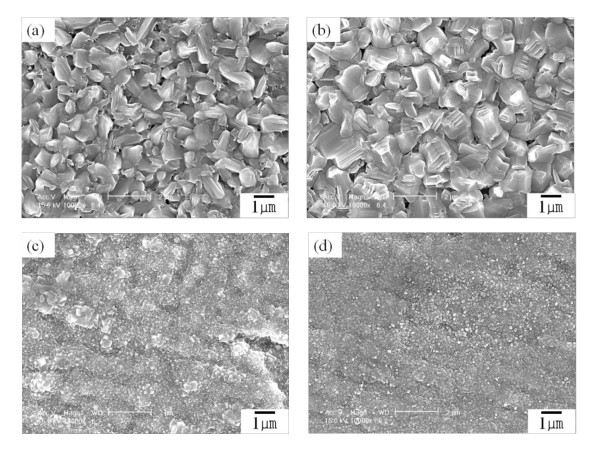
**FE-SEM images of Ni catalysts**. FE-SEM images of Ni catalysts formed on (**a**) Ti substrate, (**b**) 20-nm Au buffer layer/Ti substrate (**c**) 40-nm Au buffer layer/Ti substrate, and (**d**) 60-nm Au buffer layer/Ti substrate.

The morphology of CNTs is influenced by the catalyst particle size and density. Our experiment was thus designed to form and control the catalyst particles before CNT synthesis by NH_3 _gas pretreatment. A nickel catalyst layer was separated into well-defined discrete catalyst particles by etching of NH_3 _gas in high temperature. Also, the metal substrate in this work can be etched by NH_3 _gas. Figure 1 presents FE-SEM images showing the morphology of the Ni catalyst nanoparticles according to the thickness of the Au buffer layer after the NH_3 _gas pretreatment. In the samples with no buffer layer and a 20-nm Au buffer layer, as shown in Figure 1a, b, the surface of the substrate was very rough without noticeable particle formation, which is less favorable for CNT growth. Regarding these results, we have surmised that the absence of catalyst particles and the rough substrate surface after NH_3 _gas pretreatment are due to etching by NH_3 _gas and diffusion between the Ni catalyst layer and the Ti substrate. On the other hand, the nickel catalyst layer of the samples with Au buffer layers of 40 and 60 nm was transformed to nanoparticles, as shown in Figure 1c, d. Also, the nanoparticles showed a uniform size distribution and have an average diameter of 100 nm. From the results of Figure 1, we confirmed that an Au layer over 40 nm plays an important role in the formation of catalyst particles through suppression of interdiffusion between the Ni layer and the Ti substrate and by protection of the Ti substrate from NH_3 _gas. To investigate the effects of the Au buffer layer in CNT synthesis, CNTs were grown on a Ni/Ti substrate (no Au buffer layer) and Ni/Au/Ti substrate.

**Figure 2:**
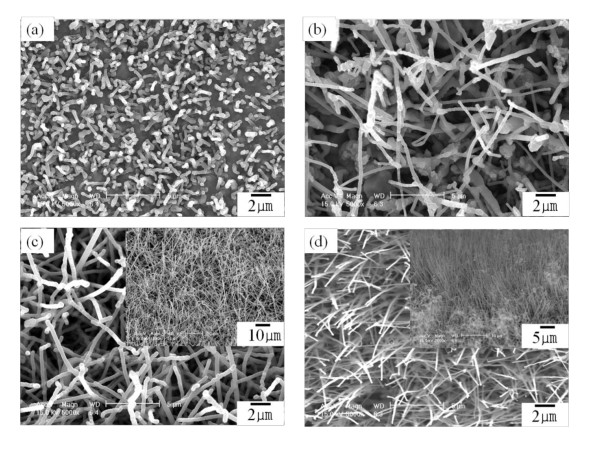
**FE-SEM images of CNTs**. FE-SEM images of CNTs grown on (**a**) Ti substrate, (**b**) 20-nm Au buffer layer/Ti substrate (**c**) 40-nm Au buffer layer/Ti substrate, and (**d**) 60-nm Au buffer layer/Ti substrate.

For CNT synthesis, four samples shown in Figure 1 were prepared. FE-SEM images of these samples after CNT growth are provided in Figure 2. Only random and sparse CNTs in the samples without and with a 20-nm Au buffer layer were obtained, as shown in Figure 2a, b. On the other hand, the samples with 40- and 60-nm Au buffer layers present growth of vertically aligned CNTs, as shown in Figure 2c, d. The results of Figure 2 accord with that of Figure 1. The insets of Figures 2c, d are low-magnified FE-SEM images. From these images, it is clear that the samples with a 60-nm Au layer have more vertically aligned CNTs. The CNTs in Figure 2d have a diameter of about 80 to 120 nm and a length of about 5 to 10 μm.

**Figure 3:**
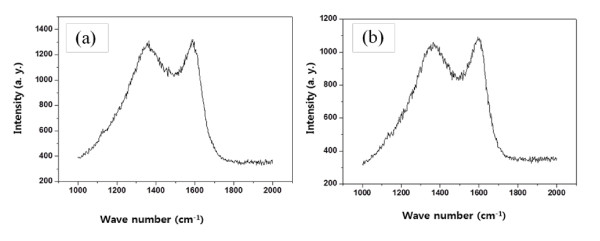
**Raman spectra of CNTs**. Raman spectra of CNTs on (**a**) 40-nm Au buffer layer/Ti substrate and (**b**) 60-nm Au buffer layer/Ti substrate.

Figure 3 shows the Raman spectra of CNTs synthesized on samples with 40- and 60-nm Au buffer layers. The Raman spectra are observed to have two prominent peaks at approximately 1,363 cm^-1 ^(noted as the D band) and approximately 1,604 cm^-1 ^(noted as the G band) with an intensity ratio *I*_D_/*I*_G _of 0.99. From the D band, which has been ascribed to disorder-induced features due to the finite particle size effect for lattice distortion, the observation that the *I*_D_/*I*_G _ratio is about 1 leads us to conclude that the CNTs have a lower degree of structural defects.

**Figure 4:**
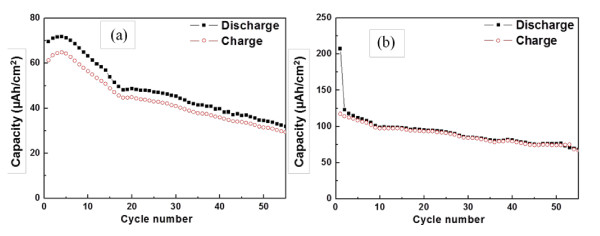
**Cycle performances of Li/CNT cells with current of 50 μA**. Cycle performances of Li/CNT cells with current of 50 μA on (**a**) Ti substrate and (**b**) 60-nm Au buffer layer/Ti substrate.

Figure 4 shows the cycle performances of Li/CNT cells with a current of 50 μA and a cutoff voltage of 0.01 to 2.0 V. Figure 4a, b present the electrochemical properties obtained using the two samples shown in Figure 2a, d as an electrode of Li/CNT cells, respectively. The electrochemical properties in Figure 4b are superior to those indicated in Figure 4a. As seen in Figure 4b, the first discharge capacity and discharge capacity after the 50th cycle were 210 and 80 μAh/cm^2^, respectively. A Li/CNT cell with CNTs on a 60-nm Au buffer layer exhibited a good charge/discharge behavior, good cycle performance, and slow capacity fading.

## Conclusions

Vertically aligned CNTs were uniformly grown on a large area of a Ni/Au/Ti substrate by a thermal chemical vapor deposition process. The CNTs prepared on an Au buffer layer with a thickness exceeding 40 nm were more uniform and abundant compared to those grown on substrates without a buffer layer or with a 20-nm Au buffer layer. A Li/CNT cell consisting of CNTs on a 60-nm Au buffer layer/Ti substrate exhibited a lower irreversible capacity and better cycle performance than a cell with CNTs on a Ti substrate. The present findings suggest that an Au buffer layer is a good candidate for a barrier of interdiffusion between the catalyst layer and metal substrate for CNT synthesis. This work also provides an enhanced approach for secondary battery electrode fabrication.

## Competing interests

The authors declare that they have no competing interests.

## Authors' contributions

YHG carried out the experimental works involving SEM, XRD, cycle testing, etc., and drafted the manuscript. JKH participated in the cycle testing. KKC participated in designing the study. HSK participated in drafting the manuscript. All authors read and approved the final manuscript.
